# Investigating the motivational behavior of pupils during outdoor science teaching within self-determination theory

**DOI:** 10.3389/fpsyg.2015.00125

**Published:** 2015-02-18

**Authors:** Ulrich Dettweiler, Ali Ünlü, Gabriele Lauterbach, Christoph Becker, Bernhard Gschrey

**Affiliations:** ^1^School of Education, Outdoor Education and Experiential Learning, Technische Universität MünchenMünchen, Germany; ^2^Chair for Methods in Empirical Educational Research, TUM School of Education and Centre for International Student Assessment (ZIB), Technische Universität MünchenMünchen, Germany

**Keywords:** outdoor teaching, science teaching, self-determination theory, motivation

## Abstract

This paper presents data from a mixed-method pilot study (*n* = 84) searching into learning psychological aspects of an outdoor science teaching program. We use data from qualitative explorations into the pupils' learning motivation during field observation, a group interview, and open questionnaires, in order to understand quantitative measures from the Self-Determination Index (SDI), and the Practical Orientation (PO) of the program. Our data suggest that lower self-regulated pupils in “normal” science classes show a significantly higher self-regulated learning motivational behavior in the outdoor educational setting (*p* < 10^−4^), and that the outdoor-teaching has generally been perceived as more practical than teaching at the normal school context (*p* < 10^−4^), irrespective of gender or school culture. We are going to provide in-depth analyses of all quantitative findings with our qualitative data and thus explain the findings logically, with respect to the direction of the statistical interpretation, and substantially, with respect to the meaning of the discoveries. We conclude that outdoor programming appears to be a suitable tool to trigger interest in science in youngsters, especially for less motivated pupils.

## Introduction and theoretical frame

### Teaching science outdoors: practical orientation and hands-on learning

Recent studies suggest, that educational programs are especially sustainably effective, if the didactic concepts are project-driven and if the learners' basic needs for autonomy, competence, and relatedness are addressed in an adequate way (Sproule et al., 2013; Dettweiler et al., 2014; Thomas and Müller, 2014). This implies the development of didactical concepts that are able to present the learning contents in an experiential manner and within a systemic context, as happens with outdoor teaching (Crompton and Sellar, 1981; Dahlgren and Szczepanski, 1998; Jordet, 1998, 2007; Dettweiler and Kugelmann, 2010; Waite, 2011; Beames et al., 2012; Bentsen and Jensen, 2012).

With respect to teaching science, one can detect that activities that are “hands-on” in nature and use technology elicit higher interest which “highlights the need to place more emphasis on the role of activity in constructing interesting learning environments, and in the meantime, suggests that student science interest could be improved by making changes to relatively easy-to-manipulate aspects of learning environments” (Swarat et al., 2012). And although prior research has shown that pupils' motivation in science classes tends to decrease during adolescence (Simpson and Oliver, 1985; Simpson and Steve, 1990; Lee and Anderson, 1993; Anderman and Young, 1994; Osborne et al., 2003), there is recent literature that suggests that school culture has a crucial influence on pupils' motivational behavior (Vedder-Weiss and Fortus, 2011). This hypothesis is also indirectly supported by Thomas and Müller (2014) who state that in grades five and six, intrinsic motivation in science is still high but that there is a decline from grade seven on. However, didactic concepts in informal schooling settings that support the pupils' autonomy in the learning process, for example in “science labs,” provoke high intrinsic motivation in pupils despite their scoring low values in science classes.

With respect to outdoor education, a recent study by Sproule et al. (2013) in the frame of self-determination theory (see below) suggests that a 12-day adventurous project-work program promotes a higher autonomy supportive climate than in the classroom context, and that intrinsic motivation, perceived competence, and a greater emphasis on task approach goal orientation was rated higher. “Furthermore as a cohort, the students reported improvements in problem solving, collaboration, and communication as a result of the project-work experience” (Sproule et al., 2013).

### Self-determination theory

Self-determination theory (SDT, Deci and Ryan, 2000) in the pedagogical context proposes that the pupils' motivational behavior is dependent on the satisfaction of certain psychological needs, i.e., the “opportunities to experience autonomy, competence, and relatedness” (Levesque et al., 2004).

According to White (1959), competence stands for the demand to control the outcome and experience mastery. Autonomy is the universal urge to be causal agents of one's own life and act in harmony with one's integrated self (Deci and Vansteenkiste, 2004). Relatedness is the universal want to interact, be connected to, and experience caring for others (Deci and Ryan, 1985, 2000, 2002; Deci and Vansteenkiste, 2004) and the better those basic needs are satisfied, the more self-regulated are the pupils' motivational behavior patterns (Deci and Ryan, 1985, 2000, 2002; Müller et al., 2007).

The learning motivational behavior is defined on a continuous scale of self-determined action. Deci and Ryan divide this scale in three different types of motivation: intrinsic motivation, extrinsic motivation, and amotivation. Both, intrinsic motivation and amotivation are not further differentiated, whereas extrinsic motivation is segmented into four types of regulation, i.e., integrated, identified, introjected, and external regulation (Deci and Ryan, 2000, 2002).

In the context of the surveys on learning motivation, however, the use of the following four regulation domains is sufficient: intrinsic regulation (InR), identified regulation (IdR), introjected regulation (IjR), and extrinsic regulation (ExR), since especially pupils of our target age are not competent to differentiate between the domains “integrated regulation” and “intrinsic regulation” (Vallerand et al., 1992; Müller et al., 2007).

**Figure 1:**
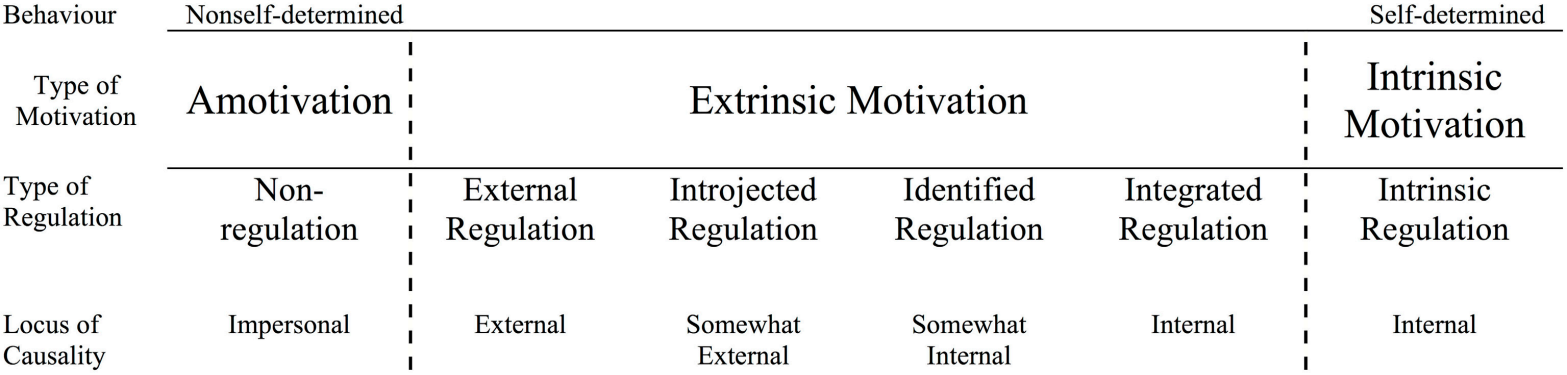
**The self-determination continuum, showing the motivational, self-regulatory, and perceived locus of causality bases of behaviors, that vary in the degree to which they are self-determined (Deci and Ryan, 2000, p. 237)**.

In the intrinsic type of regulation (InR), behaviors are measured “that are freely engaged out of interest without the necessity of separable consequences” (Deci and Ryan, 2000), in other words: actions that are “autotelic” (Csikszentmihalyi, 1975). Thus, certain behavior is regulated intrinsically if the person enjoys a particular action and likes to do it. In our study, pupils' motivational behavior is considered as intrinsically motivated if they particularly “like” or “enjoy” to learn new things. As mentioned above, intrinsic motivation is practically difficult to separate from integrated regulation where an aim is to be achieved within a particular action. However, integrated regulation “is the fullest, most complete form of internalization of *extrinsic* motivation, for it not only involves identifying with the importance of behaviors but also integrating those identifications with other aspects of the self” (Deci and Ryan, 2000, cursive added). It still remains extrinsic motivation—even though completely volitional—because it is instrumental and not autotelic. However, the vertical line at the far right end in Figure 1 divides integrated regulation from intrinsic regulation.

The type of identified regulation (IdR) describes the “direction” of behavior of a person because of his or her identification with certain aims and values. In our case, the pupils' motivational behavior is considered as “identified” if they show no particular interest in a subject but perceive the need of expertise and good grades in relation to the desired degree. “This is the process through which people recognize and accept the underlying value of a behavior. By identifying with a behavior's value, people have more fully internalized its regulation; they have more fully accepted it as their own” (Deci and Ryan, 2000).

In the domain of introjected regulation (IjR), the person acts on the basis of external social or societal expectations that were previously internalized. Thus, motivation is considered as regulated in an introjected way if a pupil learns for a subject, in order to make teachers and classmates think that she or he is a good student. This entails individuals' taking in external regulations and maintaining them in a form that is relatively isomorphic with the external regulations. The pupils

*“swallow [… ] regulations whole without digesting them. [….] Introjection represents a partial internalization in which regulations are in the person but have not really become part of the integrated set of motivations, cognitions, and affects that constitute the self. Because introjected regulations have not been assimilated to the self, the resulting behaviors are not self-determined. As such, introjected regulations are particularly interesting because these regulations are within the person, but still relatively external to the self” (Deci and Ryan, 2000)*.

In case of external regulation strategies (ExR), negative consequences such as bad grades or reprimanding by the parents are to be avoided and the students' behavior is controlled by specific external contingencies. In SDT, external regulation is considered controllin g, and externally regulated behaviors are predicted to be contingency dependent in that they show poor maintenance and transfer once contingencies are withdrawn (Deci and Ryan, 1985).

### The “research weeks” at the student science centre in berchtesgaden

The research weeks at the Student Science Centre in Berchtesgaden are one-week residential courses, where biological and climatological topics are taught under the general heading of eco-climatology (Cornelius et al., 2013; Kirchner et al., 2013). At the core of the program, a two-day research expedition is conducted into the National Park Berchtesgaden with an overnight stay at a secluded mountain hut on the base of the “Blaueis”-glacier. During the course, the students collect data in their specific field along a transection of elevation. The expedition has been prepared at their home schools, and the collected data is analyzed by the pupils in the Student Science Centre after returning from their expedition.

The program merges two educational theories—the German “Bildungstheorie” in the tradition of the “Critical Theory” of the Frankfurt-School (Becker, 2005; Bietz et al., 2005; Klafki, 2005, 2007; Adorno, 2006; Liessmann, 2006; Klafki and Braun, 2007) and the American tradition of “Outward Bound Expeditionary Learning” (Campbell et al., 1996; Bushweller, 1997; Cousins, 1999). The central concept of the German “Bildungstheorie” is the idea that education/literacy (“Bildung”) is an embodied (“leiblich;” Böhme, 2002, 2003) and aesthetic process of interaction with the world and encompasses didactic guidelines as situational-, place-based, and every-day-life relevance, person centered orientation, gender justice, and experiential learning. The corresponding didactical methods include “learning against resistance,” “learning by doing,” group, and project work, individualization of teaching processes, or “learning by teaching” (Dettweiler et al., 2011). Similar approaches can be found in recent literature (Allin and Humberstone, 2010; Humberstone and Stan, 2012; Behrendt and Franklin, 2014).

On the other hand, Outward Bound Expeditionary Learning

*“seeks to develop a deeper understanding of knowledge through the development of skills of inquiry. Students are taught to raise significant questions, to seek data, information or knowledge about those questions, and engage in inquiry in the real world. After they gain new understanding they are asked to hold up these new ideas and answers to the critique of reality” (Fischer and Mazurkiewicz, 2011)*.

In both educational theories, two concepts are taught: on the one hand, the very subject matter, in our case science (botany, geology, meteorology), and on the other hand, moral development, in order to “work with norms and values. Each experience focuses on fostering a community atmosphere, personal discovery, and personal responsibility” (Fischer and Mazurkiewicz, 2011). In the case of the research weeks, the pupils not only have to work as explorers collecting data during the expedition, but they also have to function as a team.

### Research question and guiding hypotheses

The main interest in our research design is to understand the complex of learning motivational aspects with certain didactic features of the program in the context of the outdoor educational setting during the research weeks. Data from a pre-test survey, which is not reported in this paper, suggest positive effects of the research weeks (intervention) on the pupils' motivational behavior in science lessons. So three guiding hypotheses (HG) can be formulated:

- HG_1_: The pupils' learning motivational behavior measured in the context of the research week (FoWo) is higher than at the classroom (NuT)^2^.
- HG_2_: The pupils perceive the practical orientation (PO) of the program at FoWo as higher than at NuT.
- HG_3_: Group dynamics and physical activity levels correlate positively with self-regulated motivational behavior.

Each guiding hypothesis contains a number of sub-hypotheses, which can be referred to in detail in Supplementary Table 1.

A fourth set of questions searches into cross- and gender-effects between the three different complexes. Therefore, we will not provide a set of a priori defined hypotheses but rather choose a pragmatist constructivist epistemology along the lines of “understanding phenomena” a posteriori (Biesta and Burbules, 2003).

## Methodology

### Methods of data collection

Each of the three above-mentioned hypotheses requires different methodological approaches summing up to a mixed-method approach. Data for the thematic complex of learning motivational behavior is collected by ethnographic field-notes from pupil observation (Koepping, 1987; Emerson, 2001; Bogner et al., 2002; Hammersley and Atkinson, 2007) and pupil questioning including open ended questionnaires as well as five interrogations conducted during one intervention as a group-interview (Atkinson, 1990; Flick et al., 2004). Hereby, student observation was performed on a general group level. For ethical reasons, the observed behavior has been documented anonymously. For the same reason, we did not identify the children in the group-interview, who had been randomly chosen, three girls and two boys, with their questionnaires. Those, however, had been pre-coded and personalized in a pseudo-anonymous way, so that the person looking at the questionnaires in the school- and in the outdoor-education contexts could issue the corresponding pairs of questionnaires. Data from the questionnaires are quoted in the essay with the syntax “#school, week, student number, gender.”

Quantitative measures with the German version of the Academic Self-Regulation Questionnaire (SRQ-A) originally developed by Ryan and Connell (1989), and adopted especially for our target group by Müller et al. (2007) complete the picture we aimed to gain from the pupils' learning motivational behavior.

The second complex of themes, practical orientation of the didactic concept, was searched into by issuing a questionnaire consisting of five items, each indicating a different type of practical relevance. This inventory was adopted from the scale “practical orientation” developed by Rakoczy et al. (2005) and is validated especially for our target group.

Data on complex three, group coherence and factors of well-being together with physical activity level, were gained by means of pupil observation and pupil questioning, using both, open (qualitative) and closed inquiries (ordinally scaled data). Quantitative features of the pupils' activity level have been determined by the recording of the pupils' heart rates with acentas™ heart rate monitoring devices. However, only a short but representative sample of 6:30 h, taken from two cohorts in school B has been included in the analyses.

We tested *n* = 84 pupils in three different weeks in 2013 (24; 26; 39) from two different schools, referred to as A (*n* = 20) and B (*n* = 64). All pupils were between 10 and 12 years old, and consisted of 43 girls and 41 boys. The schools differ with respect to their academic profiles. Whereas school B is a university reference school known for its science teaching, school A is a so-called “Elite School of Sports” accredited by the German Olympic Sports Confederation, and some of the pupils in school A were prospective elite athletes. Consequently, the pupils' behavior and academic performance throughout the intervention was assumedly different.

### Methods of data analysis

Data analysis was conducted according to the requisites of the given methods of data collection, including statistical analyses of quantified variables from the questionnaires, quantitative linguistic analyses, and classical content analyses for the data from interviews, field notes, and open questionnaires. In order to achieve sufficient inter-coder reliability of the qualitative interpretation of the data, two coders have applied independent coding strategies. Coder_1_ accessed the digitalized text with NVivo® live-coding (Gibbs, 2002; Lewis, 2004; Bringer et al., 2006; Bazeley, 2007), whereas coder_2_ performed “classical” content analysis on the hand-written material (Flick et al., 2004; Flick, 2007; Mayring, 2014). All authors compared the two coded sets, and the categories had been extracted as a consensus.

Coder_1_ produced an NVivo® output on the basis of the core categories, and coder_2_ re-coded the hand written material accordingly. Data are originally in German, and all interpretational operations were conducted in German on the German text. Only after the analysis, we have translated those text-chunks that are directly quoted in this paper into English for the sake of international dissemination.

The calculation of simple descriptive statistics, such as means and its graphical visualizations, allowed us a deeper interpretation of the data and indicated which groups could be reasonably compared with respect to *p*-values. We used interactive graphics to get an overview of central descriptive statistics regarding the relevant variables, and further analyses have been undertaken with Trellis-plots that give graphical visualizations in the form of matrix displays and enable easy and efficient exploration of multivariate data (Cleveland and Becker, 1996; Sarkar, 2008). Mean comparisons and One-Way ANOVAs using the more robust Brown-Forsythe and Welch test were applied to identify significances in our datasets (Garson, 2012). We tested the assumptions of One-Way ANOVA. The homogeneity of variances were examined by Levene's test, the normality assumption of the residuals could be attested by skewness, Shapiro-Wilk, and Kolmogorov-Smirnov test, as well as with appropriate diagnostic plots.

To examine the increase of Self-Determination Index (SDI) from the school to the outdoor context (diff_SDI_ = SDI_FoWo_ − SDI_NuT_), we utilized also regression techniques, such as linear regression and (orthogonal) polynomial regression. In this regard, we initially explored the monotone linear relationships among the SDI variables (SDI_NuT_, SDI_FoWo_, and diff_SDI_) by a simple scatterplot matrix. Furthermore, we computed a simple linear model, fitting diff_SDI_-values against SDI_NuT_-values, and tested the assumptions of linear regression—that are (a) linearity, (b) normal distribution of residuals (c) homogeneity of variances, and (d) outliers or influential points (leverage; Cook's distance)—by appropriate diagnostic plots (Cohen, 2010).

Reliability of the inventories have been calculated using Cronbach's α (Cronbach, 1951; Cronbach and Shavelson, 2004). Additionally, we performed a contextual analysis of quantitative features of the qualitative data from the pupil questioning with the SDI-measures in order to validate the plausibility of the findings (Mayring, 2014).

In order to search into the effects of the “during-course” factors with respect to learning motivation, we executed correlation analyses. For measuring statistical dependence between the ordinally scaled items, we used Spearman's rank correlation coefficient (ρ) and paired *t*-tests to search into differences in the separate teaching environments, indoor vs. outdoor. For the computations, we used SPSS and the open source software R (www.R-project.org).

## Findings

### Learning motivation

#### Motivational behavior from pupil observation and questioning

The analyses of the field-protocols during the research weeks show that the pupils are generally highly motivated and spirited with the assignments. They appeared to enjoy the week, worked long hours without seeming to mind and achieved great results, which they presented to the rest of the group with clearly observable pride and joy.

However, we can detect a general difference in motivational behavior between the two schools. Whereas the program could be run without disciplinary difficulties in both weeks in school B, many interventions reacting on the pupils' behavior were necessary from the instructors during the course in school A resulting from a generally observable lack of understanding of the academic side of the expeditionary teaching approach.

The group-interview conducted with school B revealed that the perceived autonomy and “freedom” in the assignments, together with being outdoors rather than in a classroom were the most important differences according to the pupils to “normal school.” Thus, one anonymous boy reports that at the research week,

“everything is much freer. You can do everything yourself. I really like it to think a little bit myself here, and that I can experiment with things.”

And one girl said that she especially liked the project character of the assignments, and that one had more time than the 45 min of a typical school lesson. The freedom of choosing methods and research approaches in the assignments was a topic mentioned by all five pupils, and was deepened in a little discussion that arose during the interview.

The field protocols show that expressions of negative and positive motivation are distributed in correspondence with the answers in the open questionnaires.

Positive motivational behavior can be observed foremost in the context of the hike with respect to movement tasks as sliding in the snow or climbing.

“We had adventure during our hike to the glacier. We climbed the steep slopes in the rain; it was a new experience. And although it was so cold up there, we really had fun at the glacier. Also the view and the sense of the forces of nature were an experience.” (#B24.29, boy)

Furthermore, the team-work and the fun during the assignment appealed to the students, too, as one girl recapitulates:

*“In my opinion it is fun and working-together that counts most because you need it for everything else” (#B39.14, girl)*,

which resulted in the matter of fact, that—as one boy put it in a short but poignant sentence:

*“we learned a lot but we learned it in a pleasant way (not like we do in school)” (#B24.24, boy)*,

Or, as one girl sums up the experience of the whole week:

*“A lot of hiking, a lot of laughing, no stress to have to get good grades, a lot of botany, tired.” (#B24.3, girl)*.

Negative expressions instead are motivated mainly by weather conditions and heave luggage, as the following two statements of a girl and a boy illustrate:

“It was exhausting because I had to carry such a heavy backpack, but I only had the absolutely necessary things with me and it was also exhausting because it was wet and cold and therefore I was not up for the whole thing as I would have been if the weather conditions were nicer.” (#A26.11, girl)

“Why do we have to take the longer route? Why can't we walk 10 minutes on a paved road instead of walking for one hour on a forest trail? And all that at a temperature of −10.000 degree Celsius in coldness and rain with backpacks that seem to weigh 10 tons and feet that hurt.” (#A26.8, boy)

Other negative motivational expressions refer also to social interactions and to strain from the hike, as can be seen from the following two anchor examples:

“For me, sleeping in one room together in the bunkhouse with ten people was stupid. We had to stay awake as long as the others were up.” (# A26.7, girl)

“It was strenuous when we had to hike up the mountain and it was also strenuous to have to act normal with every one.” (#B39.14, girl)

Finally, negative motivational expressions are, in some rare cases, also stimulated by the assignment itself or by the attending teacher, especially in school A, where we could observe tensions and a climate of mutual disrespect between pupils and one particular teacher.

However, negative expressions sometimes directly contradict some of the positive motivational utterances—reflecting the very individual experience of an objective situation. But, as the numerical output of those data show, positive utterances are by far more frequent than negative and several students claimed that they would love to come again the following year and would like to have an even deeper experience:

*“For me, this kind of action is very important. Next time I'd love to have even more.” (#B24.2, boy)*.

#### Numerical output of the qualitative data

The quantitative analysis of the data shows that 51% of all words uttered about the research week (FoWo) refer to positive motivational expressions, whereas only 8% can be classified as negative motivational expressions (cf. Table 1).

**Table 1:**
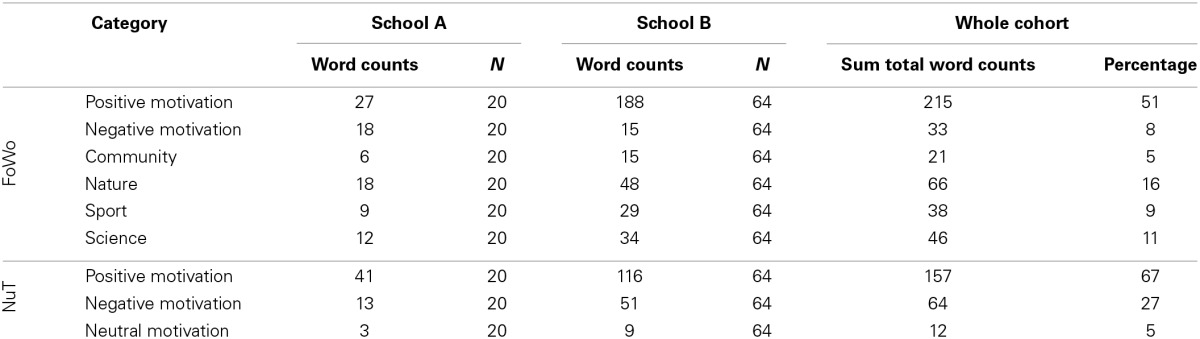
**Word counts by category**.

**Figure 2:**
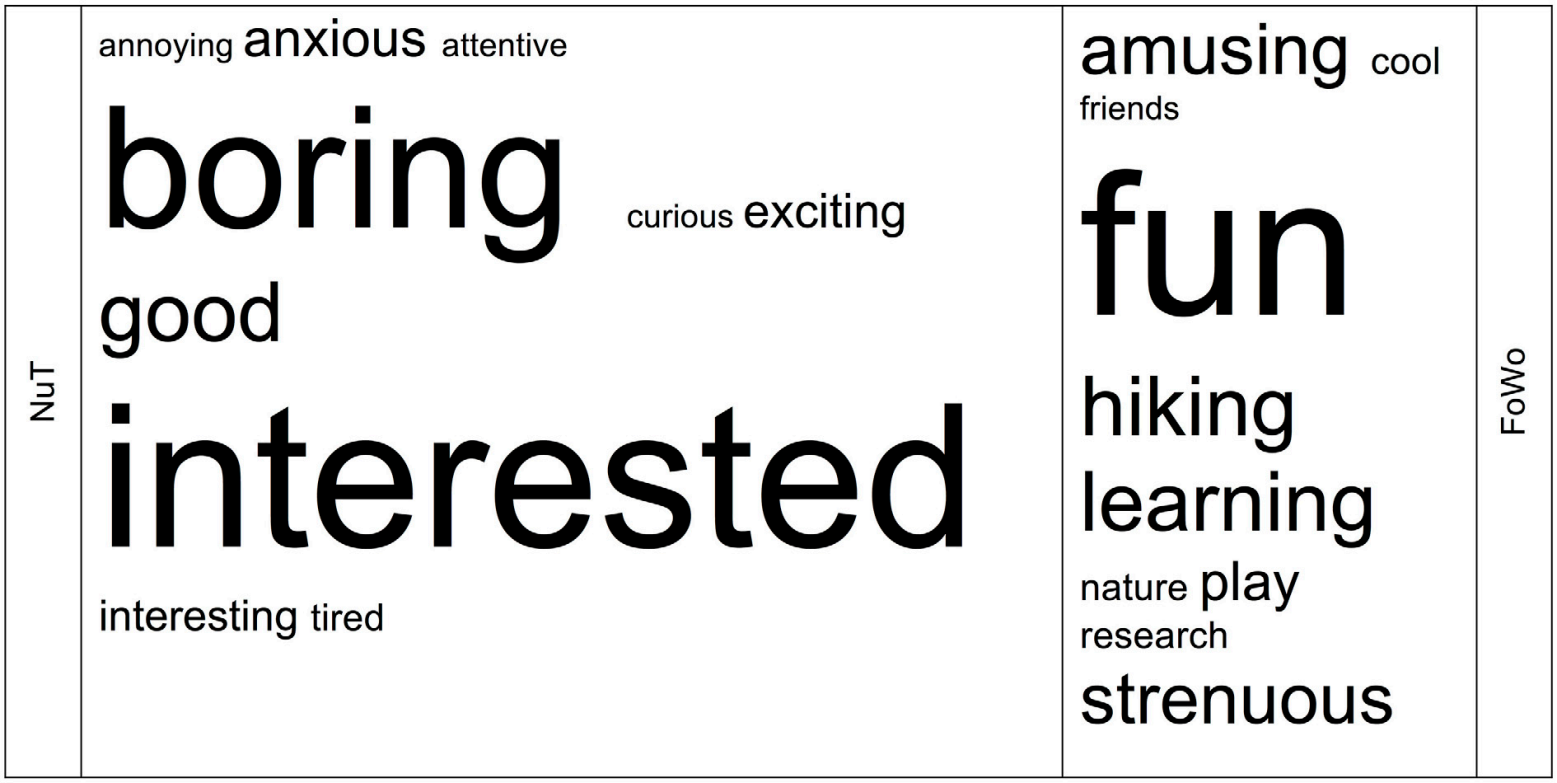
**Comparisons of the 10 most frequently used words in the contexts of NuT and FoWo. Greater font-size indicates higher word-frequency in the questionnaires**.

Looking at the word frequencies, one can see that “fun” is with 39 counts by far the most used word, followed by “learning” and “hiking” (each 18 counts). If we add “fun” and “amusing” (16 counts)—two very similar concepts, at least in their German origin (“Spaß” and “lustig”)—it becomes very clear what has been the prevalent emotional impression for the pupils during the research week (cf. Figure 2).

When we look at the science lessons at school (NuT), we can detect fewer categories—naturally, due to the different frame, words in such categories as “nature” or “sport” do not appear, but interestingly, we also do not find any references to “science.” The relative frequency of words with respect to positive motivation is 67%, that of words with connotation to negative motivation is 27%, fairly higher than at the research week. Five Percent of the words in the NuT-context can be classified as expressions of “neutral motivation.”

When we look at the words directly, we can see that “interested” and “boring” are almost equally frequent distributed followed by “good,” “anxious,” and “exciting.” It thus becomes clear that the emotions associated with lessons at school are more ambiguous and certainly more negative than at the research week.

#### SDI-values – some quantitative explorations

In addition to the qualitative data, we used the SDI (Deci and Ryan, 2000) in order to quantify the pupils' motivational behavior and to compare those values in the two teaching settings statistically. The SDI is calculated from the abovementioned four motivational domains, intrinsic motivation (InR), identified motivation (IdR), introjected motivation (IjR), and extrinsic motivation (ExR):

SDI=(2 · InR+IdR) − ( IjR+2 · ExR).

A simple descriptive output of the pupils' SDI-values in the two different teaching settings (SDI_FoWo_, SDI_NuT_), as well as the arithmetic difference of the two variables (diff_SDI_ = SDI_FoWo_ – SDI_NuT_), reveals interesting results.

**Figure 3:**
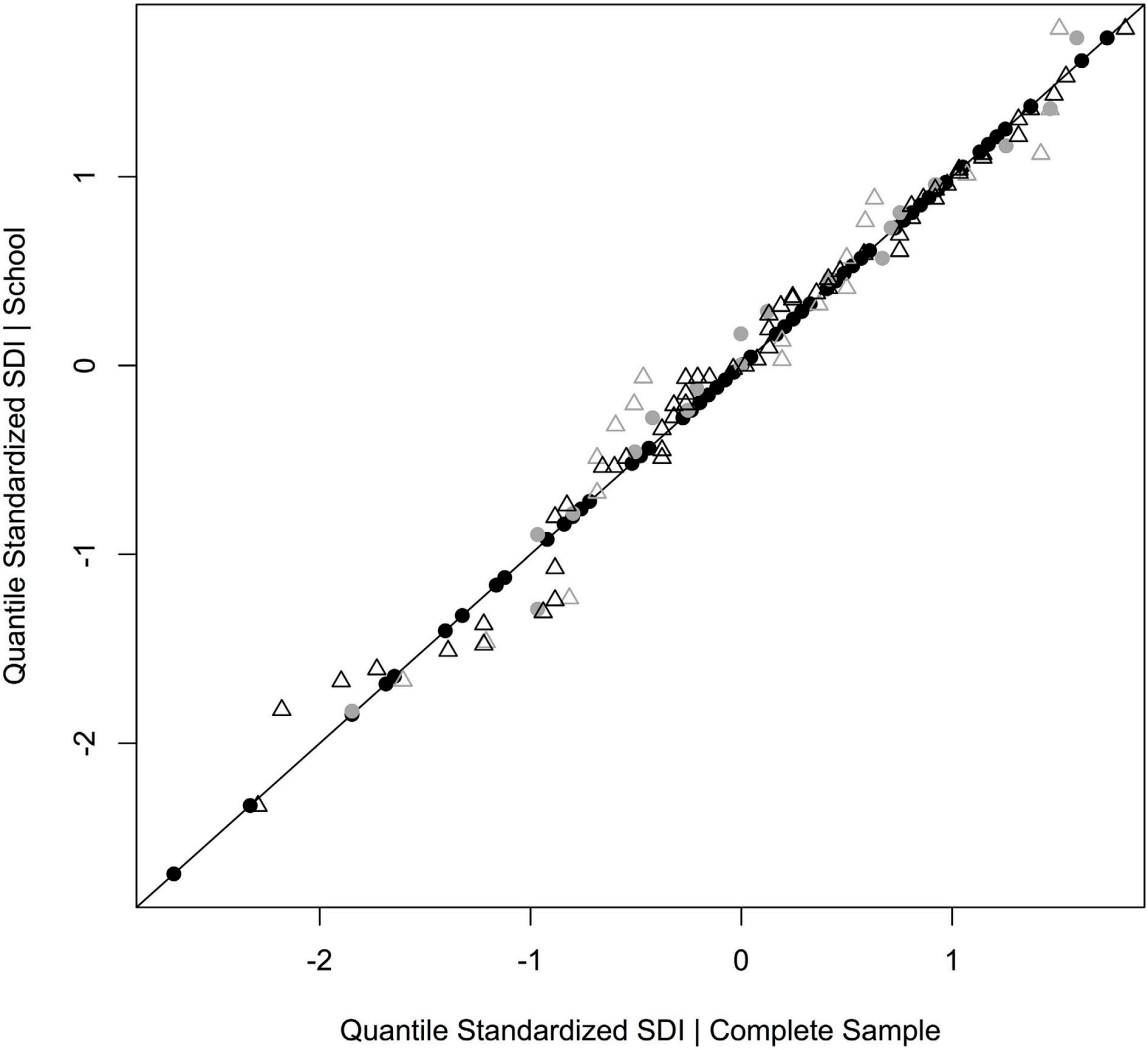
**Displayed are the quantiles of standardized SDI-values conditioned on school (y-axis) plotted against the quantiles of standardized SDI-values computed in the complete sample (x-axis), in order to compare their probability distributions**. Black symbols refer to school B, gray symbols to school A. Circles refer to NuT-values, triangles to FoWo-values. We can see that the data set as a whole and the parts of it split by school are approximately equally distributed regarding the quantiles and corroborate robust results in both teaching contexts.

First of all, we can see that the SDI-mean values during the research weeks are considerably higher than in the classroom—indicating higher self-regulated motivational behavior in the outdoors than in the classroom. With respect to the school comparison, **Table 3** shows that pupils differ significantly in their motivational behavior, both in the indoor and in the outdoor contexts (*p* < 10^−4^). This somewhat extreme difference between the groups, however, does not debauch the data set as a whole, as can be seen from Figure 3.

**Figure 4:**
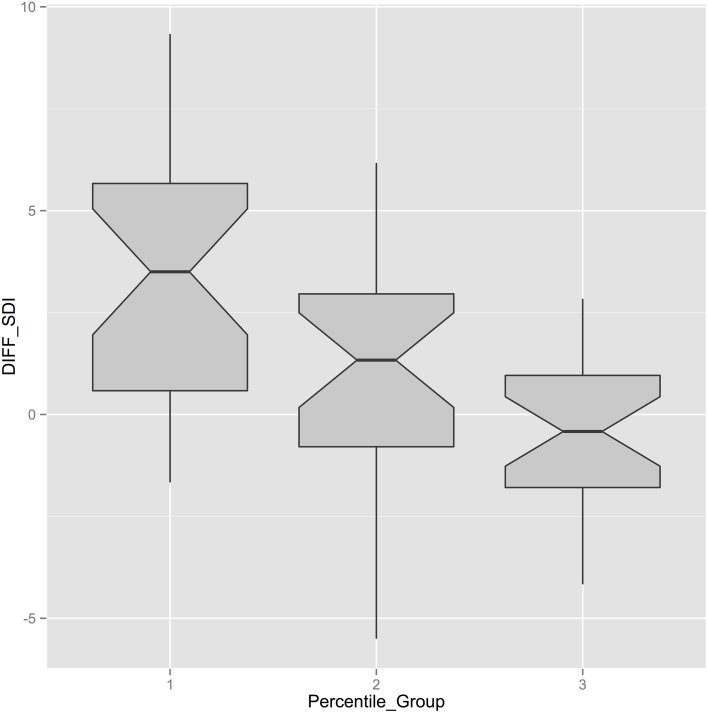
**Boxplots displaying the difference in SDI-measures in FoWo and NuT (diff_SDI_) with respect to the three percentiles in the classroom**. It can clearly be seen that the difference in SDI is considerably higher in the first percentile of SDI_NuT_ and is constantly decreasing with the higher thirds.

**Figure 5:**
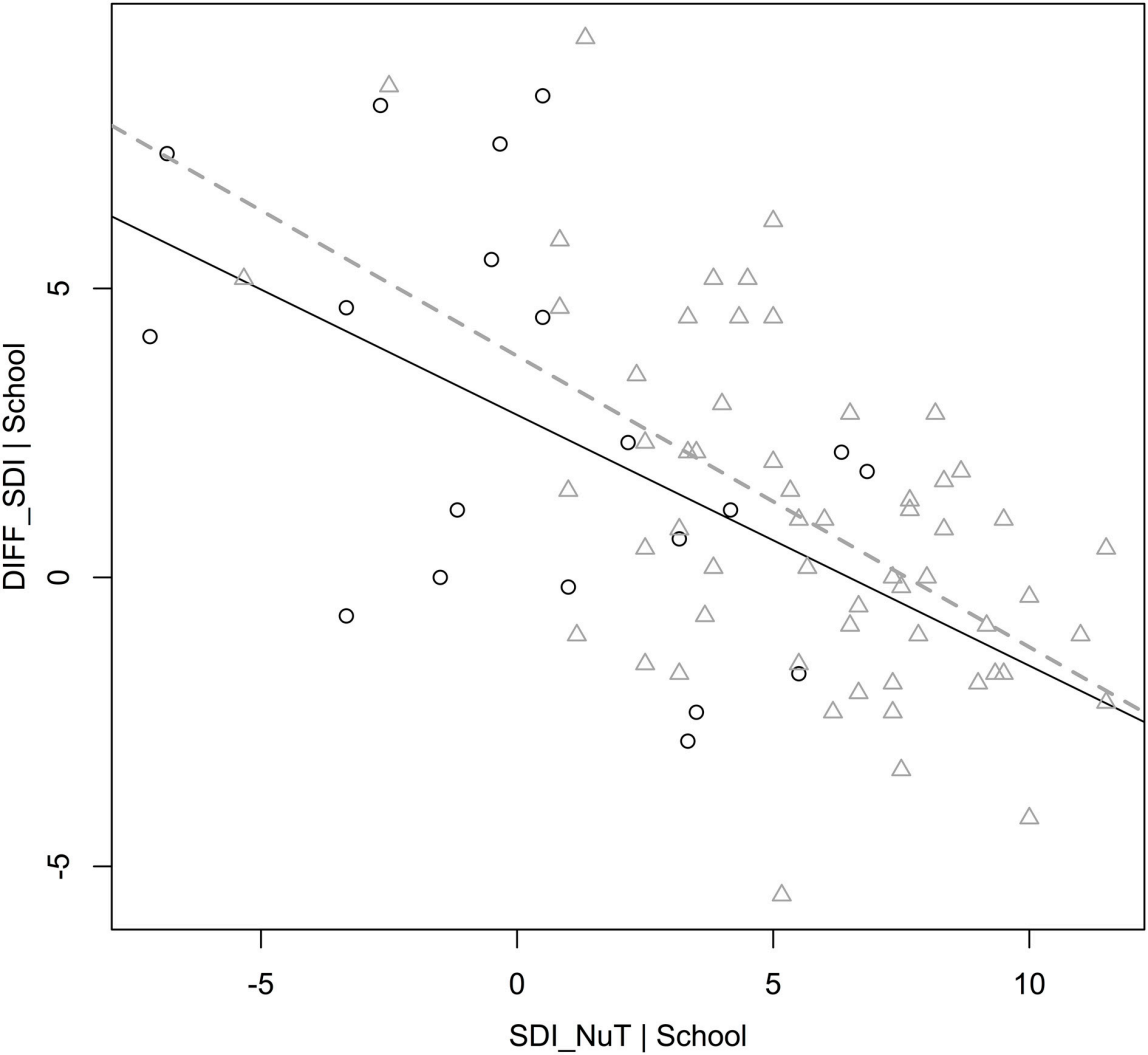
**Displayed are the regression lines of the diff_SDI_-values with respect to both schools**. The dashed line resembles the behavior of pupils in school B based on the data points marked with triangles. The straight line represents the behavior of pupils in school A based on the data points marked with circles. One can clearly see that irrespective of the relative “entrance” level of SDI_NuT_, the pattern of a monotone decreasing, linear function of the two variables can be obtained. This means that differences in school culture may affect the degree of self-regulated motivational behavior but not the fact that especially less self-regulated pupils in NuT profit from outdoor science teaching.

**Figure 6:**
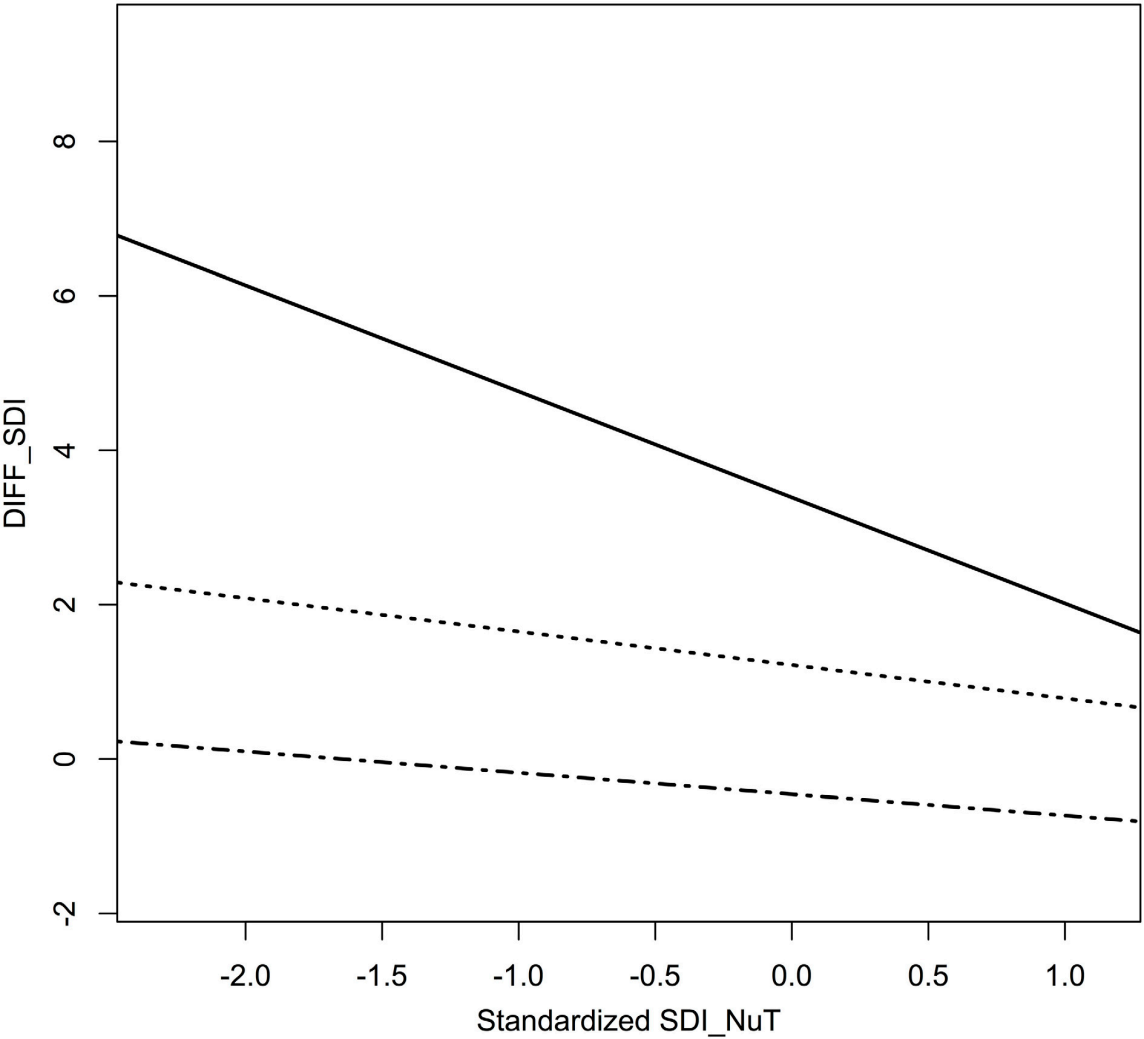
**Displayed are the differences in SDI based on standardized SDI_NuT_-values in the three percentiles**. The above curve (full line) represents the first third (0–33%) of SDI_NuT_, the middle curve (dotted) the second third (34–66%), and the lowest curve (full-dotted) represents the third percentile (67–100%). It can be seen that the biggest difference or near-constancy in motivational behavior occur in the extremes of lowest and highest SDI_NuT_-values, respectively. That means on the one hand, that pupils who are exceptionally extrinsically motived in the classroom, achieve the highest relative increase in intrinsic motivation during the outdoor teaching session, and on the other hand, that pupils, who are highly self-regulated in science class at school, show a merely slightly more extrinsically motivated behavior in the outdoors, albeit that change is relatively small and negligible as compared to the first third of lowest motivation values.

Now, if we look at the percentiles with respect to motivational behavior in the classroom (SDI_NuT_), i.e., construe three equal groups in ascending motivational behavior starting with the least self-regulated kids, and plot their SDI_NuT_-values against the difference in motivational behavior (diff_SDI_) as the dependent variable, we can see that those pupils who are least self-regulated in class achieve the highest increase of SDI during the research week with peaks up to 9.34 points on a scale of 24, mean diff_SDI_ = 4.26 (cf. Figure 4). Those pupils, who show already very high self-regulated motivational behavior in class, behave practically equally highly motivated during the research week (mean diff_SDI_ = −0.35, cf. Figure 5). It is interesting to see that boys and girls do equally well-respond to the expeditionary learning treatment, but that factors with respect to school culture seem to influence the degree of self-regulated motivational behavior but not the general pattern of a stronger response of less intrinsically motivated pupils to the intervention (cf. Figure 6).

We can clearly support the guiding hypothesis HG_1_ and claim that the pupils' learning motivational behavior measured in the context of the research week (FoWo) is significantly higher than at the science lessons at school (NuT) and that those pupils, who are less self-regulated at NuT profit especially from the outdoor teaching with respect to self-regulated learning (cf. Tables 2–6).

**Table 2:** **Mean scores and standard deviations for SDI_FoWo_ and SDI_NuT_**.

			**Gender**				**School**			
			**Male**		**Female**		**School A**		**School B**	
			**SDI**_**FoWo**_***: n = 41***		**SDI**_**FoWo**_***: n = 43***		**SDI**_**FoWo**_***: n = 20***		**SDI**_**FoWo**_***: n = 64***	
			**SDI**_**NuT**_***: n = 38***		**SDI**_**NuT**_***: n = 41***		**SDI**_**NuT**_***: n = 20***		**SDI**_**NuT**_***: n = 59***	
	**Mean**	**SD**	**Mean**	**SD**	**Mean**	**SD**	**Mean**	**SD**	**Mean**	**SD**
SDI_FoWo_	5.76	3.53	5.47	3.42	6.04	3.61	2.87	3.80	6.66	2.93
SDI_NuT_	4.34	4.13	4.21	3.80	4.45	4.45	0.51	3.97	5.64	0.51

**Table 3:** **ANOVA: SDI_FoWo_, SDI_NuT_ (factor: school)**.

		**ANOVA (*School*)**				
		**Sum of squares**	***df***	**Mean square**	***F***	**Sig**.
SDI_FoWo_	Between groups	218.52	1	218.52	21.99	0.000
	Within groups	814.78	82	9.93		
	Total	1033.31	83			
SDI_NuT_	Between groups	392.64	1	392.64	32.33	0.000
	Within groups	935.23	77	12.15		
	Total	1327.88	78			

**Table 4:** **ANOVA: SDI_FoWo_, SDI_NuT_ (factor: gender)**.

		**ANOVA (*Gender*)**				
		**Sum of squares**	***df***	**Mean square**	***F***	**Sig**.
SDI_FoWo_	Between groups	6.85	1	6.85	0.55	0.462
	Within groups	1026.46	82	12.52		
	Total	1033.31	83			
SDI_NuT_	Between groups	1.11	1	1.11	0.06	0.801
	Within groups	1326.77	77	17.23		
	Total	1327.88	78			

**Table 5:** **Descriptives: diff_SDI_ (factor: Percentile groups of SDI_NuT_)**.

	**N**	**Mean**	**Std. Deviation**	**Std. Error**	**95% confidence interval for mean**		**Minimum**	**Maximum**
					**Lower bound**	**Upper bound**		
1	27	3.3200	3.37784	0.65007	1.9838	4.6562	−1.67	9.34
2	26	1.0450	3.03196	0.59462	−0.1796	2.2696	−5.50	6.17
3	26	−0.4550	1.73402	0.34007	−1.1554	0.2454	−4.17	2.83
Total	79	1.3289	3.18957	0.35885	0.6144	2.0433	−5.50	9.34

**Table 6:** **ANOVA: diff_SDI_ (factor: percentile groups of SDI_NuT_)**.

	**Sum of squares**	***df***	**Mean square**	***F***	**Sig**.
Between groups	191.876	2	95.938	12.119	0.000
Within groups	601.645	76	7.916		
Total	793.522	78			

#### Reliability testing of the SDI-inventory

The reliability testing of the SDI-inventory needs some attention, since we adopted the inventory, and as to our knowledge, used it for the first time in an outdoor educational context.

We tested Cronbach's α for both, the inventory as a whole and separately for the four different motivational domains InR, IdR, IjR, and ExR in both teaching settings (cf. Table 7). We also performed a factor-analysis and re-calculated the SDI-values after having excluded one critical item from the inventory, and compared the re-calculated SDI-values with the original values. The comparison shows that the original SDI-values corroborate much more stable SDI-values, despite the inclusion of a critical item. Put together this suggests that our SDI-values are robust and reliable.

**Table 7:** **Reliability based on Cronbach's α**.

	**InR**	**IdR**	**IjR**	**ExR**
α_FoWo_	0.77	0.58	0.68	0.28^a^
α_NuT_	0.84	0.66	0.43	0.61

a The critical value 0.28 for ExR_FoWo_ can be accounted for by means of factor-analyses and the test-wise exclusion of the critical item #10, which yields α_ExR___FoWo_ = 0.61. However, mean comparisons with excluded item #10, SDI-values show even greater differences between FoWo and NuT. We therefore decided to calculate SDI-values including item #10 in order to stabilize the findings by putting more stress on the p-value.

Furthermore, the consistency of the SDI-measures are backed by a modified split-half-test of the qualitative data (Mayring, 2014). But instead of splitting our qualitative data-set in two random halves, as Mayring suggests (p. 107), we chose to split the data by the group “school” which yields differences in SDI-measures. Since we found similar patterns in the quantitative features of the qualitative data as in the SDI-values, we assume a high level of validity in the qualitative data and reliability in the SDI-values (cf. Table 8). However, we are very aware of the criticism articulated against such an approach in content analytical analyses, since

*“[w]ith reliability determination, parallel testing procedures appear problematic, as the equivalence of two instruments used for analyzing language material is likely to be demonstrable only in rare cases. The splitting method is also unlikely to be appropriate in most instances, since the size of the material sample, as also the size of the instrument (categories), is mainly defined in such a way that in individual parts central findings can occur which alter the overall results” (Mayring, 2014)*.

**Table 8:** **Validity quotient (Q_V_) based on word-count-index (I_WC_)**.

**Category**	**Group 1 (School A)**			**Validity quotient (Q_*V*_)**	**Group 2 (School B)**		
	**Word counts**	***N***	$I_{WC\_A}=\frac{\sum Word\;count}{N}$	$Q_{V}=\frac{I_{WC\_B}}{I_{WC\_A}}$	$I_{WC\_B}=\frac{\sum Word\;count}{N}$	***N***	**Word counts**
Positive motivation	27	20	1.4	2.1^a^	2.9	64	188
Negative motivation	18	20	0.9	0.2^b^	0.2	64	15
Community	6	20	0.3	0.7^c^	0.2	64	15
Nature	18	20	0.9	0.9^c^	0.8	64	48
Sport	9	20	0.5	1.0^c^	0.5	64	29
Science	12	20	0.6	0.8^c^	0.5	64	34

a If we compare Q_V_ of positive motivation (2.1) to the ratio of SDI_FoWo_ of School A and SDI_FoWo_ of School B (2.3), we can clearly see a similar frequency pattern, which supports the validity of the modified split-half test and the data set as a whole.

b There is no comparative value from the SDI-inventory to estimate negative motivation (nota bene: “extrinsic motivation” is not the same as “negative motivation”). However, the explainable difference of positive motivation between the groups suggests also a difference in negative motivation, i.e., a number considerably smaller than 1 for Q_V_, which is clearly the case (Q_V_ = 0.2 < 1).

c In those categories, we would expect similar frequencies among the two groups, which yields an expected Q_V_ = 1. Our results show ratios between 0.7 and 1.0, which again supports the validity of the modified split-half test and adds plausibility to the findings.

However, in our specific case, the quantitative features of our language material appear to be appropriate and add plausibility to our quantitative findings.

### Practical orientation of the didactic concept

#### Findings from the PO-inventory

The overall practical orientation of the program is calculated by simply adding up the mean values of the five items. The overall PO-mean value for NuT is 12.92, that for FoWo is 15.26. Thus, we can retain hypothesis HG_2_ and can, by means of a simple *t*-test, even show that the difference in means is highly significant (*p* < 10^−4^).

The overall Cronbach's α for the five items is 0.69 for the context of FoWo (*n* = 84, valid *n* = 78), and 0.84 for NuT (*n* = 84, valid *n* = 78). According to general conventions, those values are fairly reliable and need no further argumentation in that respect.

When we tested hypotheses item by item of the inventory by means of paired-samples tests, items 1, 2, 3, and 5 show significant differences with greater values during the research week (*p* < 0.005). This means that the pupils find that

- in the outdoor context, general rules could be better deferred from practical examples than at NuT,
- in the outdoors, the general applicability of the learned was better than at NuT,
- the developing of new knowledge during the outdoor teaching took off more from own experiences than at NuT, and
- in the outdoors, the specific applicability of the learned with respect to every-day-life-problems was better than at NuT (cf. Supplementary Table 1).

Only the reference to every-day-life examples during lessons at school and during the research week showed no significant differences.

By looking closer at the results of the paired-samples test, one can see that the difference of means of item 2, i.e., the general applicability of the learned, is constantly significant throughout the percentiles of SDI_NuT_ and gender. That means that irrespective of gender or learning motivational behavior at school, the pupils found what they learned at the research week generally more applicable. No other patterns in the distribution of significant *p*-values of the paired-samples *t*-tests of the other items can be detected throughout the percentiles and gender (cf. Table 9).

**Table 9:** **Pairwise comparison of didactical concepts in the NuT and FoWo contexts**.

**Paired samples test**						
		**Mean**	***SD***	***T***	***Df***	**Sig. (two-tailed)**
Pair 1	“practical examples”	2.57	0.93	−2.97	78	0.004
		2.97	1.00			
Pair 2	“general applicability of the learnt”	2.62	0.84	−6.55	78	0.000
		3.35	0.83			
Pair 3	“starting from own experiences”	2.58	0.93	−3.88	78	0.000
		3.11	0.86			
Pair 4	“every-day life examples”	2.77	0.97	−0.35	78	0.728
		2.82	0.92			
Pair 5	“specific applicability of the learnt”	2.33	1.05	−4.80	77	0.000
		2.94	0.92			

#### Observations of textual features from the open questions concerning practical orientation

Furthermore, it is interesting to see that in the classroom context, not a single word in the questionnaires can be attributed to “science” or any content-related category, whereas 11% of all relevant words fall into that category in the outdoor-context. By looking at the pupils' comments, one can see that observations being categorized as “science” are situations with mainly positive motivational attributes:

“The hike was very strenuous but really enjoyable! I did not like that we had to measure sooo often! But in the end, it was worthwhile the effort!” (#B39.26, girl)

“For me, nature is the most important thing. I connect the hike with nature. It

was a good idea that everyone in the botany group had her own special plant. It was a lot of fun to take such a close look at nature and to classify all the plants and then of course there was this breath-taking view from the mountain hut and from the glacier.” (#B24.8, girl) “I was in the botany group. We explored plants. In the mountains and in the valley. Thanks to the measurement tools we were able to discover many things.” (#B39.11, girl) “… and when I dissected the blossoms it was super-interesting to actually be able to see how such a flower looks from the inside.” (#B24.2, girl)

Although there was one exception: weather conditions. Here we can see that the pupils complain about their tasks during heavy rain, for example for pupil #B24.3, boy, who said that “the rain was annoying, especially on the glacier.” But in the end, those expressions were relatively rare and the pride of the achievement of having been on a glacier at not so good weather conditions seems even stronger, than the situational complaint according to the motto: Well, everyone can hike up a mountain in good weather conditions, but we did it although it was pouring down!

A seemingly strong effect on the practical orientation of the outdoor-program was the construction of measurement tools, at least for the “weather group.” The notes from a reflection round in week 24 show that the building of their own tools is connoted with very positive motivational expressions—which can also be understood as an influencing factor to the high presence of PO-related subjects in the analysis of learning motivation.

“I really enjoyed hiking to the mountain hut and building our own measurement tools was very funny and exciting.” (#B24.26, girl)

### Group coherence and physical activity level as influencing during-course factors

**Figure 7:**
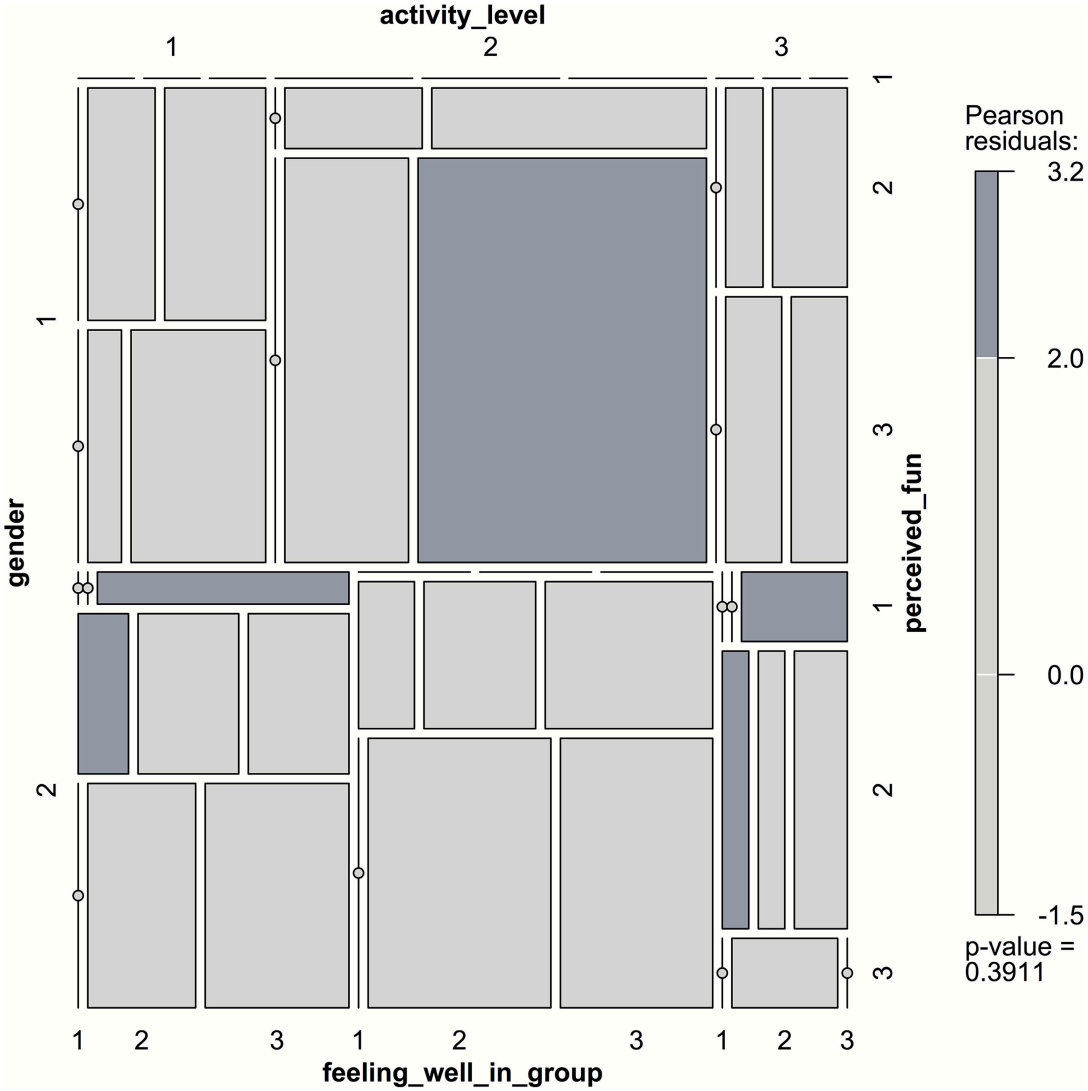
**Mosaic-plot indicating some during-course factors of the expedition**. Tile areas are proportional to the frequencies of cross-tabbed variables. The darker fields indicate a relatively stronger deviation from an assumed independence model, with Pearson residuals and overall *p*-value shown on the right. In particular, it can be seen that girls (gender = 1) at “moderate” activity level (2) expressed to have found it “fairly” (2) or “very enjoyable” (3) during the expedition and felt “fairly well” (2) or “very well” (3) in the group. This is in accordance with our observations of group behavior during the expedition. Girls chose a moderate pace at certain neuralgic stages during the hike (i.e., steep sections, sections short before a goal etc.), stayed together in crowds, and chatted actively (Dettweiler et al., 2014). However, a difference between boys and girls regarding those variables cannot be deemed statistically significant (*p* = 0.39).

As can be seen from the Figure 7, most pupils felt very well in the group during the research week. As we have learned from the textual data, the pupils have found new friends and had a feeling of great fellowship during the expedition. When asked what word comes to their mind when they reflect the research week, one girl answered:

“Companionship: we really were a very great group in which I felt totally at ease. One could ask anyone anything and one was able to talk with actually everyone. Moreover, no one was excluded from the group altogether!!!” (#B24.7, girl)

The outdoor setting also seems to be a good way to overcome prejudices and to get to know other students:

“I am now good friends with some girls from my school class that I had not so much in common with before!” (#A26.10, girl)

“I have found new friends! I knew 2 of the girls from before as we have PE-lessons together and I really did not like them. But during this research week I have been sharing the room with them and 2 other girls I did not know before and we all have become great friends and got along very well.” (#B24.13, girl)

Data from the quantitative measures of group coherence and physical activity level indicate that there are no significant differences between boys and girls (*p* = 0.39). However, one pattern can be detected from the numbers: especially girls at “moderate” activity level expressed to have found it “fairly” or “very enjoyable” during the expedition and felt “fairly well” or “very well” in the group (cf. Figure 7). This is in accordance with our observations of group behavior during the expedition. Girls chose a moderate pace at certain neuralgic stages during the hike (i.e., steep sections, sections short before a goal etc.), stayed together in crowds, and chatted actively. This corresponds with objective physical activity parameters measured in two cohorts in school B. One can clearly see that the pupils' activity level is relatively high during the hike, with maximum heart rates of 197 bpm (boys) and 189 bpm (girls), and an average heart-rate of 122 bpm (girls), respectively 113 bpm (boys) over several hours, adding up to an extra metabolic work-load of ca. 700 kcal/day in the field. It is also interesting to see that the activity behavior of boys and girls differs significantly during the hike at certain points (*p* < 0.005). The boys' activity level goes significantly up at two times—firstly short off the big break at the “Cake Alp,” a very steep section, secondly short off the final destination of the day. The girls seem to keep rather a steady pace, very constant with respect to the mountain-guides in the control group (Dettweiler et al., 2014). However, the pupils' subjective evaluation of the physical activity level in the questionnaires does not reveal significant differences with respect to gender (*p* = 0.49).

Furthermore, we do not see any big differences either with respect to lower- or higher self-motivated children in school either. Words categorized as “community,” cannot be found in the reports about the school context, against 33 nominations (8%) in the outdoor-context.

Apart from the companionship, “fun” was really what made the difference for the kids. As long as they enjoyed themselves, everything else just came along—or as someone said:

“The best thing was actually the hiking as we learned something and had fun.” (#B.26.16, girl)

We can state that about 17% of all pupils report that it was “very strenuous,” and half of the group found it still “fairly strenuous”—but most pupils found it “fairly” or even “very enjoyable.” Only two pupils (two boys, #A26.20 and #B39.21) did not like the hike at all and found it “not enjoyable.”

Part of the joy seems to derive from the impressive nature around them. In the field notes there is one description of a boy who stands on the terrace of the mountain hut early in the morning and looks down into the valley. When asked how he felt, he said that now, as he takes in this view, he knows it was really worth while the hardship during the hike of the day before. And there are many more statements about the landscape to be found in the texts:

“The nature around us was really beautiful and exciting (glacier). Hiking and climbing in the evening with the mountain guides was really great. Sliding in the snow also was very funny §miley.” (#2B4.24, boy)

Apart from the beautiful scenery, pupils often referred to the fun in getting the chance to try out different movements when they were up at the mountain hut and got the opportunity to experience walking in loose gravel, snow or climbing big stones:

“The hike was really strenuous in the beginning, but after the ‘Cake Alp’ I had a lot of fun and the glacier was really great and the introduction to walking in snow was very funny—especially the snowball fight—and the hike down was not bad at all, it was totally exciting.” (#B24.26, girl)

It seems that despite the relatively highly perceived grade of physical activity level, the overall well-being of the pupils during the hike is not affected.

### Cross-effects and correlations

#### Practical orientation and SDI_FoWo_

Correlating the mean-values of the five items of practical orientation and SDI_FoWo_, one can see that item 1, which refers to rules that can be deferred from practical examples, positively correlates with SDI_FoWo_ (ρ = 0.278^**^, *p* = 0.006), as well as item 2, which refers to the general applicability of the learned (ρ = 0.288^**^, *p* = 0.005), and item 5, which refers to the specific applicability of the learned in everyday life (ρ = 0.192^*^, *p* = 0.044).

Moreover, the semantic analysis of the word counts of the open questionnaires shows that the pupils use a lot of verbs (68) to describe FoWo whereas no verbs are used at all for the description of NuT. Verbs are normally used to describe active processes whereas nouns are used for static relations (Langacker, 1987). The most frequently used verbs in the outdoor context are “to research” (10 counts), “to learn” (18 counts) and “to hike” (20 counts).

#### Group-coherence/physical activity level and SDI_FoWo_

Correlating the mean-values of the group-coherence factors during FoWo and SDI_FoWo_, one can see that those pupils who had especially much fun during the learning expedition show highly self-regulated motivational behavior (ρ = 0.475^**^, *p* < 10^−4^). A similar relation can be determined between those pupils who felt especially well-within the group (ρ = 0.199^*^, *p* = 0.035). We cannot detect significant differences with respect to gender.

Furthermore, we cannot show any significant correlations of group dynamical factors and diff_SDI_ either, which would allow us to indirectly determine influential factors to SDI_FoWo_. However, data from pupil observation hint that generally, in the extra-mural school setting, the combination of feeling a strong sense of community, being physically active while “learning,” and leaving the every-day learning patterns behind are aspects that can explain the increment of self-regulated motivational behavior. Here is one quote that represents a fair number of similar ones:

“For me, it was especially important that I had fun during the lessons—since then one is better motivated for learning. … It was different from everyday life at school: we hiked up to the glacier ‘during school time.”’ (#B24.5, boy)

## Summary and discussion

The most obvious finding in this survey is clearly that in the outdoor educational setting, pupils show significantly higher learning motivational behavior—irrespective of gender or school culture, but that school culture in terms of relatedness has a significant influence on the level of self-regulated motivational behavior. Moreover, less self-regulated pupils profit more from the outdoor setting than those who show already a high intrinsic learning regulation—independent from the relative SDI-level, as the school comparison shows.

This corresponds well-with recent comparative didactical research conducted in the context of student laboratories. Thomas and Müller (2014) investigated into differences of regular science classes and science labs, regarding the pupils' perceived autonomy support, intrinsic motivation, and identified regulation. Their theoretical frame was identical with our survey within SDT, using the same psychometric scale SRQ-A (Müller et al., 2007). They report that in grades five and six perceived autonomy support and intrinsic motivation were high both in science classes and in science labs. At grades seven and eight perceived autonomy support and intrinsic motivation were still high in science labs but not in science classes.

With our data, we can fully support those findings for the high values in science class with the younger age group. We have not yet searched into older pupils and will address this theoretically very interesting problem in our next surveys. However, when we compare the mean-values for intrinsic and identified regulations of boys and girls in the context of student labs and the outdoor educational setting, we can concede that both girls and boys show even a higher intrinsic motivational behavior in the outdoors than the girls and boys in Thomas and Müller's survey in the student labs. The same pattern can be detected with identified regulation. In the context of the outdoor teaching, both, girls and boys show higher values than the boys and girls at the research lab. Even if we have not directly measured perceived autonomy, as Thomas and Müller have, we can explain this pattern by theorizing that there is a linear progress of perceived autonomy from class via student labs (mostly one-day interventions) to our research weeks in the outdoors, where there is a lot of time for the pupils to engage in the research tasks and perceive competence and autonomy.

This is well-supported by our finding that the outdoor-teaching has been perceived by the pupils as being generally more practical than the teaching at the normal school context. There, “practical” stands indirectly for the two basic needs of autonomy and competence (Rakoczy et al., 2008) and promotes the connection of science (class) to every day life experiences. The latter is hypothesized as “not yet taken fully advantage of” in student labs and science classes by Thomas and Müller (2014).

If we look at the pupils' behavior during the outdoor-research, and also consider the qualitative feedback we received in the questionnaires, we can easily understand that the pupils enjoyed the consequent practical orientation of the science teaching and express to have had great “fun,” by doing things themselves (e.g., building their own measurement tools and develop their own data-collection strategy). Additionally, they perceived themselves as competent (and got instant feedback, when their self-made measurement tool showed the same humidity as the pro-tool in their backpack)—and these seem to be the main influential factors for the highly self-regulated motivational behavior. Again, we cannot account for any gender effects with respect to the variables of practical orientation of the program which accords to findings of Engeln (2004), Brandt (2005), and Guderian (2007), and this “might be a promising starting point for reducing the gender gap in the motivation for science and for winning over young people to careers in the sciences” (Thomas and Müller, 2014).

Moreover, in addition to the didactically outstanding work being done at student laboratories, the physical activity in the outdoors (hiking) and the group dynamics in the one-week residential outdoor setting can be seen as factors that contribute to the pupils' exceptionally high self-regulated learning motivational behavior. This accords with findings in Denmark, where pupils who are taught outside the classroom show significantly higher activity levels than those who are not (Mygind, 2007), and that the outdoor teaching leads to better social relations and experiences with teachers (Bentsen et al., 2009).

Furthermore, we have seen that the “fun” and “enjoyment” inherent in the hiking and the practical orientation inherent in the scientific research projects add to the already stimulating pedagogical setting. This is in accordance with Bisson and Luckner (1996) who searched into the pedagogical role of fun in adventure education and found that “fun can have a positive effect on the learning process by inviting intrinsic motivation, suspending one's social inhibitions, reducing stress, and creating a state of relaxed alertness.”

In addition to the research previously reported looking into gender effects, we also compared four levels of internal regulation among the pupils and found that especially lower self-regulated pupils in “normal” science classes show a significantly higher self-regulated learning motivational behavior in the outdoor educational setting. This may well be invoked by the physical activity inherent in the outdoor field research, a factor that is suggested to be specifically helpful for kids with attention deficits in the ordinary school context (Gapin and Etnier, 2010; Verret et al., 2012). Even if we have not tested the lower self-regulated pupils for any attention deficit disorder, and do not suppose highly external regulation as an indicator for any attention deficit syndrome, we could theorize that similar activity treatment patterns do their work here. Further research with well-defined criteria concerning (the different types of) ADHD and external motivational behavior would have to prove this hypothesis.

All together, these findings suggest that it is worthwhile to intensify efforts to develop concepts for autonomy promoting and competence imparting “hands-on”-teaching science. The outdoors seems to be a perfect starting point to promote this didactical approach in the schools.

However, the factors being responsible for an increase of self-regulated learning motivation need to be determined more precisely and with better research tools, and seem to depend much on school culture, as the school comparison shows. Our findings suggest that relationships between the pupils and between pupils and teachers also add into that—which confirms SDT with respect to the basic need of relatedness, as well as stressing factors at school and during the outdoor experience. Therefore, the Basic Psychological Needs Questionnaire (Deci and Ryan, 2000) as well as the Learning Climate Questionnaire (Black and Deci, 2000) seem to be most promising and appropriate tools (Sproule et al., 2013). Additionally, the monitoring of the pupils' physical activity in the outdoors is not yet totally satisfying (Mygind, 2007; Dettweiler et al., 2014), and we have addressed this problem in a follow-up study by applying new technologies for activity monitoring (Türmer et al., 2013) and the inclusion of neurological measurements, such as amygdala activity by means of MRT scans (Lederbogen et al., 2011) as well as cortisol and cfDNA probes as stress indicators (Breitbach et al., 2012; Mierau et al., 2014). We expect to correlate physical activity levels with motivational behavior more concisely than we are able to at the moment and will link that also to the research domain of cognitive behavior under physical exercise (Tomporowski, 2003; Coles and Tomporowski, 2008; Tomporowski et al., 2008).

This leads to another complex of research desiderates, i.e., the quality of the learned contents during the outdoor teaching in comparison to the “normal” school context in a longitudinal design, i.e., content quantity and quality, as well as learning sustainability, which has been searched into in a mixed-method approach by Christie et al. (2013), yielding promising results. In our above-mentioned longitudinal survey, we will also address this problem in a control group design.

That the expeditionary outdoor teaching has the potential to make a deeper impression on at least some of the kids, is reported exemplarily by the teachers of one school, where several pupils have started their own afternoon science groups after having returned from the research weeks doing research and signing up for student research competitions.

In this sense, we want to conclude our paper with referring to a quote of one boy that stands for many:

*“We have learned a lot of new things we did not know before! Thanks!” (#B26.2, boy)*.

### Conflict of interest statement

The authors declare that the research was conducted in the absence of any commercial or financial relationships that could be construed as a potential conflict of interest.

## Footnotes

^2^“FoWo” is short for German “Forscherwoche”, “research week” (outdoor). “NuT” is short for German “Natur und Technik”, “nature and technology” (indoor).

## Supplementary material

Supplementary Table 1 **Hypotheses**.
