# Supplementary material for: Investigating the motivational behavior of pupils during outdoor science teaching within self-determination theory
Source: Front Psychol. 2015 Feb 18;6:125. doi: 10.3389/fpsyg.2015.00125 (PMC4331641; doi:10.3389/fpsyg.2015.00125)
Supplement: Supplementary Table 1 — Hypotheses. [file Table1.DOCX]

Table 1. Hypotheses

| **HG1: The pupils’ learning motivational behaviour measured in the context of the research week (FoWo) is higher than at the classroom (NuT).** | **HG_2_: The practical orientation of the programme at FoWo is perceived higher by the pupils than at NuT.** | **HG3: Group dynamics and physical activity levels are relevant factors for positive motivational behaviour.** |
| --- | --- | --- |
| HS_1.1_: The pupils’ learning motivational behaviour at FoWo is more self-regulated than at NuT. | HS_2.1.1_: At FoWo, the pupils find that general rules can be better deferred from practical examples than at NuT. | HS_3.1_: There is a positive correlation between self-regulated motivational behaviour and well-being within the group. |
| HS_1.2_: Those pupils, who are less self-regulated at NuT profit especially from the outdoor teaching with respect to self-regulated learning. | HS_2.1.2_: At FoWo, the pupils find the general applicability of the learned better than at NuT. | HS_3.2_: There is a positive correlation between self-regulated motivational behaviour and enjoyment of the expedition. |
|  | HS_2.1.3_: At FoWo, in the perception of the pupils, the developing of new knowledge takes off more from own experiences than at NuT. | HS_3.3_: There is a negative correlation between enjoyment of the expedition and the perceived exercise-level of the expedition. |
|  | HS_2.1.4_: At FoWo, in the perception of the pupils, the reference to every-day-life examples is higher than at NuT. | HS_3.4_: There is a negative correlation between the perceived exercise-level of the expedition and self-regulated motivational behaviour. |
|  | HS_2.1.5_: At FoWo, the pupils find the specific applicability of the learned with respect to every-day-life-problems better than at NuT . | HS_3.5_: There is a positive correlation between the perceived enjoyment of the expedition and self-regulated motivational behaviour. |
|  | HS_2.2.1_: At FoWo, there is a positive correlation between the pupils perception, that general rules can be deferred from practical examples, and highly self-regulated motivational behaviour. |  |
|  | HS_2.2.2_: At FoWo, there is a positive correlation between the general applicability of the learned and highly self-regulated motivational behaviour. |  |
|  | HS_2.2.3_: At FoWo, there is a positive correlation between the developing of new knowledge, which takes off more from own experiences, and highly self-regulated motivational behaviour. |  |
|  | HS_2.2.4_: At FoWo, there is a positive correlation between the reference to every-day-life examples and highly self-regulated motivational behaviour. |  |
|  | HS_2.2.5_: At FoWo, there is a positive correlation between the specific applicability of the learned with respect to every-day-life-problems and highly self-regulated motivational behaviour. |  |
